# Evaluation of serum levels of sestrin 2 and betatrophin in type 2 diabetic patients with diabetic nephropathy

**DOI:** 10.1186/s12882-024-03663-2

**Published:** 2024-07-19

**Authors:** Asmaa Mounir Emara, Amal Said El Bendary, Laila Mahmoud Ahmed, Hanaa Ibrahim Okda

**Affiliations:** 1 Internal Medicine Department, Faculty of Medicine, Tanta University, Tanta, Egypt; 2 Clinical Pathology Department, Faculty of Medicine, Tanta University, Tanta, Egypt

**Keywords:** Sestrin 2, Betatrophin, Type 2 diabetes, Diabetic nephropathy

## Abstract

**Background:**

Diabetic kidney disease (DKD) is one of the most serious microvascular complications of diabetes mellitus (DM) and the leading cause of chronic kidney disease (CKD) worldwide. Since obesity and type 2 DM (T2DM) are considered as inflammatory conditions, thus reducing their accompanied systemic inflammation may lessen their complications. Sestrin 2 belongs to a group of stress induced proteins which are produced in response to oxidative stress, inflammation and DNA damage. Betatrophin; a hormone that stimulates the growth, proliferation and mass expansion of pancreatic beta-cells and improves glucose tolerance. The objective of the study was to evaluate levels of serum Sestrin 2 and betatrophin in patients with different stages of diabetic nephropathy (DN)) and compare results with healthy control.

**Methods:**

This cross sectional study was carried out on 60 patients above 18 years old, recruited from Tanta University hospitals out patients clinics and 20 apparently healthy individuals of matched sex and age as a control group. Participants were divided into two groups: group I: 20 normal subjects as control group and group II: 60 patients with type 2 DM,. further subdivided in to three equal groups: group 1IIA(20 patients) with normo-albuminuria (ACR < 30 mg/g), group IIB (20 patients) with micro albuminuria (ACR = 30 to 300 mg/g) and group IIC (20 patients) with macro albuminuria (ACR > 300 mg/g). They were subjected to detailed history taking, careful clinical examination and laboratory investigations including blood urea, serum creatinine, estimated glomerular filtration rate (eGFR), urinary albumin creatinine ratio, and specific laboratory tests for Sestrin 2 and Betatrophin by using ELISA technique.

**Results:**

Serum Sestrin 2 significantly decreased, while serum betatrophin level significantly increased in macroalbuminuric group compared to control and other 2 diabetic groups (*P* value < 0.05). The cut off value of serum sestrin 2 was 0.98 ng/ml with sensitivity 99%, specificity 66% while the cut off value of serum betatrophin was > 98.25 ng/ml with sensitivity 98%, specificity 82%. Serum betatrophin positively correlated with age, fasting, 2 h postprandial, BMI, triglyceride, total cholesterol, serum creatinine, blood urea, UACR, and negatively correlated with eGFR and serum albumin. Serum Sestrin 2 positively correlated with serum albumin. BMI, serum urea, UACR and serum albumin. Serum betatrophin are found to be risk factors or predictors for diabetic nephropathy.

**Conclusions:**

Patients with DN, particularly the macroalbuminuria group, had a significant increase in betatrophin levels and a significant decrease in serum Sestrin 2 level. The function of Sestrin 2 is compromised in DN, and restoring it can reverse a series of molecular alterations with subsequent improvement of the renal functions, albuminuria and structural damage.

## Introduction

Obesity and Type 2 Diabetes are regarded as inflammatory diseases, and reducing the systemic inflammation that goes along with them may help both ailments and lessen their consequences [1]. Diabetic nephropathy (DN) is one of the most deeply feared complications of diabetes. A startling 45% of T1DM and T2DM diabetics are affected by this microvascular complication [2]. Diabetes is currently the primary cause of end-stage renal disease (ESRD) in the west and become considered the main cause for patients need renal replacement therapy globally [3]. Clinically, DN is typically defined by gradual increases in urinary albumin excretion (UAE), accompanied by an increase in blood pressure and risk of cardiovascular complications. Alongside this, eGFR gradually decreases and eventually advances to ESRD [4]. At the macroalbumiuric stage of DN, the degree of albuminuria and proteinuria is a significant clinical predictor of the rate at which the disease progresses [5].

Sestrin 2, a highly conserved protein that is triggered by inflammation, oxidative stress and DNA damage, is a part of the Sestrin family which include also Sestrin1 and Sestrin3. It preserves the integrity of cells under stress through metabolic processes that generate energy and stimulation of the DNA repair system [6]. Sestrin2 expression is induced by ATP deficiency and hypoxia [7]. Reduction of intracellular levels of Sestrin2 can lead to a variety of unfavorable consequences such as mitochondrial dysfunction, oxidative damage and insulin resistance [8]. Moreover, many studies have found that Sestrin2 is also involved in atherosclerosis [9], DN [10], and other chronic vascular diabetic complications [11]. However, controversial findings were found when Sestrin 2 levels were monitored in obese and/or T2DM patients [12]. Nevertheless, it is still unclear from the data whether serum Sestrin2 levels and diabetic nephropathy are related.

The newly discovered circulating protein betatrophin, also referred to as lipasin, angiopoietin-like protein (ANGPTL8), refeeding induced fat and liver (RIFL), and chromosome 19 open reading frame 80 (TD26), is mostly secreted from the human liver. Additionally, it has been well established that betatrophin is a novel regulator of lipid metabolism in both human and rodents [13].

According to previous studies, mice’s islet 𝛽-cell proliferation was linked to high betatrophin levels [14] but knockout of betatrophin failed to impair the glucose profiles of mice [15]. Observational studies on humans have shown a correlation between circulating betatrophin and several health disorders, including T2DM [16]. However, researches have shown conflicting results on the relationship between betatrophin level and T2DM [17]. So, the purpose of this study was to elucidate the possible relationship between the serum levels of betatrophin and Sestrin 2 and the progression of type 2 DN patients.

## Patients and methods

This cross sectional study was carried out on 60 patients with T2DM (group II), aged above 18 years old and 20 apparently healthy individuals of matched sex and age as a control group (group I). The study was carried out from January to August 2023, after approval from the Ethical Committee Tanta University Hospitals and was in accordance with the principles of the Declaration of Helsinki II. An informed written consent was obtained from all patients. Patients with type 1 diabetes mellitus, type 2 diabetes with either active infection, any systemic or endocrinal diseases, heart failure, liver diseases, renal diseases other than diabetic nephropathy and pregnant or lactating female patients were excluded. Type 2 DM was diagnosed according to the American Diabetes Association criteria [18]. Diabetic patients were treated with either oral hypoglycemic agents or insulin. Group II were subdivided according to urinary albumin creatinine ratio (UACR) to 3 equal groups, group IIA included 20 patients with normo albuminuria (UACR < 30 mg/g), group IIB included 20 patients with micro albuminuria (30 ≤ UACR < 300 mg/g) and group IIC included 20 patients with macro albuminuria (UACR > 300 mg/g). All patients were subjected to full history taking with special attention paid to duration of diabetes, any drugs received and thorough clinical examination including body mass index (BMI) that was calculated by dividing patient weight in kilograms by their height in meters squared.

Laboratory investigations were performed in Clinical Pathology Departments, Faculty of Medicine, Tanta University and included complete blood count (CBC), urinary albumin creatinine ratio, blood urea, serum creatinine, fasting and postprandial blood glucose, HBA1C, serum cholesterol and triglycerides, liver function tests. Overnight fasting blood samples were withdrawn under complete aseptic technique with disposable sterilized plastic syringes. Following an overnight fast, 10 milliliters of venous blood were drawn from each participant. Two milliliters was placed in a K3 EDTA Vacutainer tube for HbA1c estimation by colorimetric method as percentage of total hemoglobin (Stanbio Glycohemoglobin, Boerne), and the remaining quantity were divided into two parts; 4 ml into plain tubes for serum separation, used for colorimetric determination of fasting (8–12 h) and 2 h post prandial blood glucose (2 h after eating ameal), triglycerides (TG), total cholesterol (TC), levels using commercial kits (Spinreact, Spain) [19], Serum creatinine was measured by jaffe kinetic technique through fully automated spectrophotometry chemical analyzer konelab 60 thermo fisher. The remaining serum was kept in aliquotsat − 80 °C till the assay of the two markers. Estimated glomerular filtration rate was calculated by CKD EPI equation (GFR = 141 * min(Scr/κ,1)α * max(Scr/κ, 1)-1.209 * 0.993Age * 1.018 [if female] * 1.159 [if black] [20]. Single-spot second morning fresh urine specimens were collected in sterile cups from all subjects, then immediately centrifuged. Urinary albumin concentration was determined in fresh spot urine samples by immunoturbidimetric method (BioSystems, Spain), Urine creatinine was measured by the same technique of serum creatinine after dilution 50 times, and the result obtained was multiplied by 50 (the dilution factor) then UACR (mg/g) was calculated. The UACR was calculated by dividing urinary albumin concentration (mg) by urinary creatinine concentration (g). two out of three measurements over the previous 3 to 6 months should fall in the macroalbuminuric or microalbuminuric range.

Serum Sestrin 2 levels was measured by the Sestrin 2 ELISA DL Develop Kit, china Catalog No: DL-SESN2-HU with detection range 0.156-10ng/mL and ELISA kits Sensitivity was 0.094ng/ml while serum betatrophin measured by Human Angiopoietin-like protein 8/Betatrophin ELISA SunRed Kit, china Catalogue No 0.201-12-5327 with detection range 7.5ng/L→2000ng/L and ELISA kits Sensitivity was 7.332ng/L.

### Statistical analysis

Statistical analysis was done by SPSS v27 (IBM©, Chicago, IL, USA). Shapiro-Wilks test and histograms were employed to assess the normality of the distribution of data. Quantitative parametric data were analyzed using the ANOVA (F) test and the post hoc Tukey test. The data were presented as mean and standard deviation (SD). Quantitative non-parametric data were compared between each group using the Mann Whitney test and the Kruskal-Wallis test. The data were presented as the median and interquartile range (IQR). The Chi-square test was used to analyse the frequency and percentage (%) of the qualitative variables. Data correlation was done using Pearson’s correlation coefficient (r) test. Receiver operating characteristic (ROC curve) analysis was used to find out the overall predictivity of parameter in and to find out the best cut-off value with detection of sensitivity and specificity at this cut-off value. A statistically significant result was defined as a two-tailed *P* value less than 0.05.

## Results

Eighty participants were involved in the study, they were divided into 2 groups: group I, included 20 apparently healthy individuals of matched sex and age who served as a control group and group II included 60 diabetic patients who further categorized based on their albumin creatinine ratio to group IIA included 20 patients with normo albuminuria (ACR ≤ 30 mg/g), group IIB included 20 patients with micro albuminuria (ACR 30 to 300 mg/g), and group IIC included 20 patients with macro albuminuria (ACR > 300 mg/g), 35 males (43.75%) and 45 females (65.25%) were participated in the study, group I had 9 males (45%) and 11 females (55%), group IIA had 8 males (40%) and 12 females (60%), group IIB had 6 males (30%) and 14 females (70%) and group IIC had 12 males (60%) and 8 females (40%), with non-significant differences between all groups (*P* value = 0.30). The mean age was 44.75 *±* 5.35 years (range 27–49 years) in group I, 46.93 *±* 4.59 years (range from 35 to 54 years) in group IIA,46.73 *±* 5.79 years (range from 35 to 54 years) in group IIB and 49.93 *±* 6.59 years (range 45–57 years) in group IIC. with non-significant differences between all groups (*P* value = 0.18) as shown in Table 1.

The mean BMI in group I was 29.12 *±* 2.3, 29.42 *±* 3.46 in group IIA, 32.9 *±* 3.36 in group IIB and 33.8 *±* 7.76 in group IIC, with significant differences between group I and group IIA (*P* value < 0.05), group A and group IIC (*P* value < 0.05) and group IIA and IIC (*P* value < 0.05). The mean duration of diabetes was 6.84 *±* 2.81 years in group IIA, 8.25 *±* 2.65 years in group IIB and 8.35 *±* 3.25 years in group IIC with non-statistically significant differences between groups (*p* value 0.51) as shown in Table 1.

The mean fasting blood glucose level was 76.4 + 9.2 mg/ dl in group I, 139.8 *±* 32.3 mg/dl in group IIA, 147.4 *±* 35.2 mg/dl in group IIB and 149.8 *±* 36.3 mg/dl in group IIC with statistically significant differences between group I group II, group I and IIA, group I and IIB and group I and IIC (*P* < 0.05). The mean level of 2 h post prandial blood glucose level was 111.27 *±* 8.8 mg/ dl in group I, 204.66 *±* 44.2 mg/dl in group IIA, 225.27 *±* 48.8 mg/dl in group IIB and 214.26 *±* 44.2 mg/ dl in group IIC with statistically significant differences between group I and group II, group I and IIA, group I and IIB, group I and IIC and group IIB and IIC (*P* < 0.05). The mean HBA1C was 7.25 *±* 1.36% in group IIA, 9.06 *±* 1.37% in group IIB and 9.76 *±* 1.37% in group IIC with statistically significant difference between group II A and II B (*p* value 0.04) and between group II B and IIC (*p* value 0.04) as shown in Table 1.

The mean total cholesterol level was 104.27 *±* 27.8 mg/dl in group I, 152.66 *±* 34.2 mg/dl in group IIA, 161.27 *±* 38.8 mg/dl in group IIB and 179.26 *±* 34.2 mg/dl in group IIC with statistically significant difference between group I and group II, group I and Group IIA, and group I and group IIC (*P* value < 0.05). The mean triglyceride level was 75.4 *±* 13.2 mg/dl in group I, 168.8 + 52.3 mg/dl, in group IIA, 136.4 + 53.2 mg/dl in group IIB and 179.8 + 43.3 mg/dl in group IIC with statistically significant difference between group I and group II, group I and Group IIA, group I and group IIC, group I and Group IIB and group IIB and IIC (*P* value < 0.05) as shown in Table 1.

The mean blood urea level was 28.1 *±* 6.2 mg/dl in group I, 28.8 + 7.3 mg/dl in group IIA, 28.7 + 6.2 mg/dl in group IIB and 55.8 + 13.3 mg/dl in group IIC with statistically significant difference between group I and group IIC, group A and Group IIC, and group II B and group IIC(*P* value < 0.05).The mean serum creatinine was 0.87 *±* 0.14 in group I, 0.86 *±* 0.16 mg/dl in group IIA 1.04 *±* 0.24 mg/dl in group IIB and 1.27 *±* 0.21 mg/dl in group IIC with statistically significant difference between groups I and group IIA, group I and group IIC, group I and group IIB and group IIB and group II C (*P* value < 0.05).The mean eGFR was 104.02 *±* 12.85 ml/min/1.73m^2^ in group I, 94.08 *±* 12.2 ml/min/1.73m^2^ in group IIA, 76.02 + 20.8 ml/min/1.73m^2^ in group IIB and 65.28 *±* 16.2 ml/min/1.73m^2^ in group IIC with statistically significant difference between groups I and group IIA, group I and group IIC, group I and group IIB and group IIB and group II C (*P* value < 0.05).The mean urinary albumin creatinine ratio was 3.5 + 1.9 mg/g in group I, 9.6 + 7.4 mg/g in group IIA, 91.8 + 55.9 mg/g in group IIB and 371.5 + 55.9 mg/g in group IIC with statistically significant difference between group I and group IIA, group I and group IIC, group I and group II B, group IIA and Group IIC, and group IIB and group IIC (*P* value < 0.05).The mean serum albumin was 4.1 *±* 0.50 g/dl in group I, 4.2 *±* 0.42 g/dl in group IIA, 3.6 + 0.52 g/dl in group IIB and 3.2 + 0.24 g/dl in group IIC with statistically significant difference between group I and group IIA, group I and group IIC, group I and group II B, and group IIB and group IIC (*P* value < 0.05) as shown in Table 1.

The mean serum Sestrin 2 level was in 2.32 *±* 0.55ng/ml in group I, 1.62 *±* 0.36 ng/ml in group IIA, 1.02 *±* 0.18 ng/ml in group IIB and 0.55 *±* 0.29 ng/ml in group IIC with with statistically significant difference between group I and group II, group I and group IIA, group I and group IIC, group I and group II B, and group IIB and group IIC (*P* value < 0.05) as shown in Table 1.

The mean serum betatrophin in 132.2 *±* 31.6 ng/l group I, 251.6 *±* 36.26 ng/l in group IIA, 307.20 *±* 31.16 ng/l in group IIB and 409.2 *±* 115.32 ng/l in group IIC with statistically significant difference between group I and group II, group I and group IIA, group I and group IIC, group I and group II B, and group IIB and group IIC (*P* value < 0.05) as shown in Table 1.

**Fig. 1:**
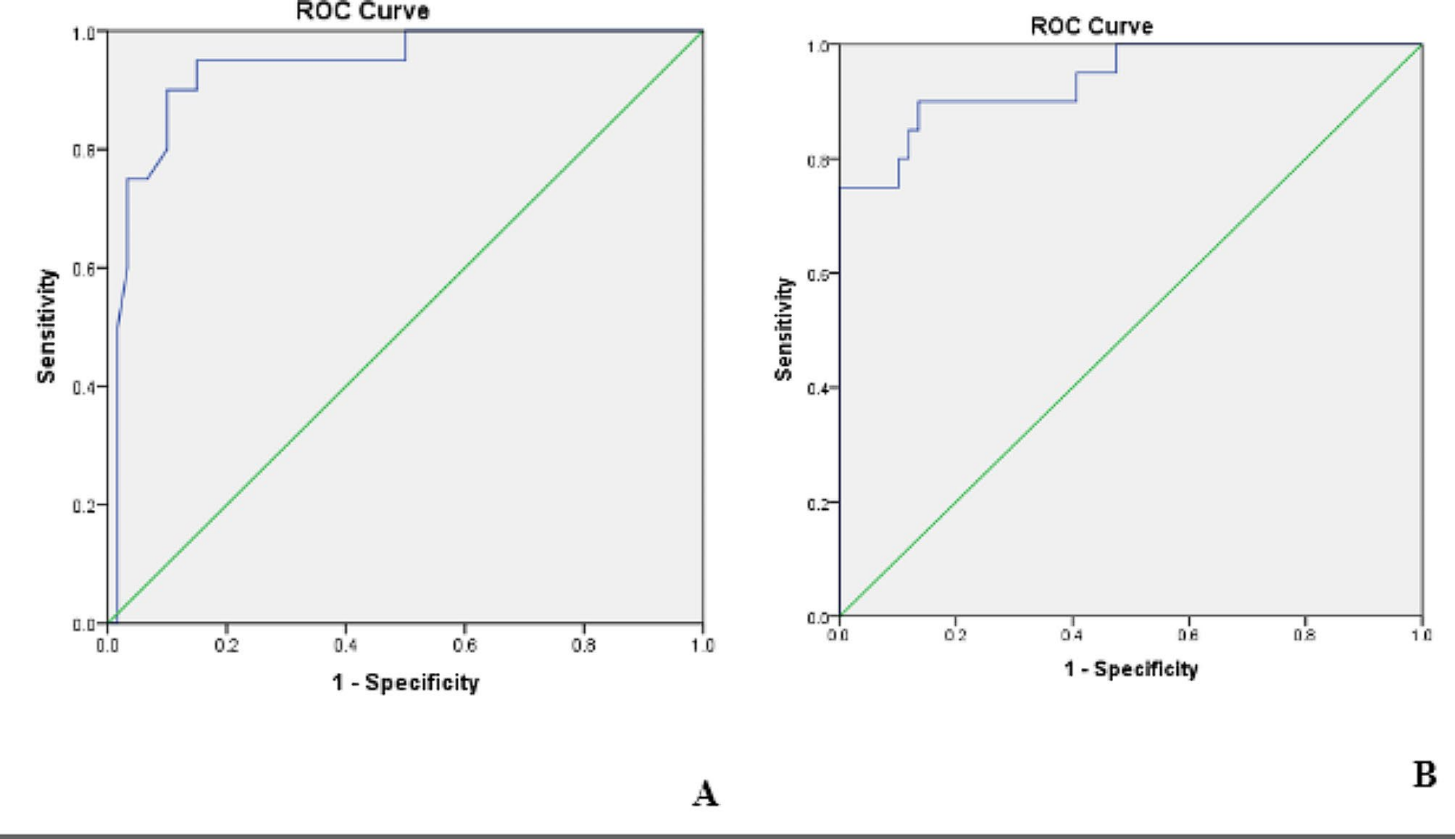
Sensitivity and specificity among patients with DN of (**A**) serum Sestrin 2 and (**B**) Serum betatrophin

ROC curve was used to evaluate the sensitivity and specificity of Sestrin 2 and betatrophin for detection of DN. The cut off value of serum Sestrin 2 to predict DN was 0.98 ng/ml with sensitivity 99%, specificity 66% while the cut off value of serum betatrophin was > 98.25 ng/ml with sensitivity 99%, specificity 82%. (Fig. 1)

**Fig. 2:**
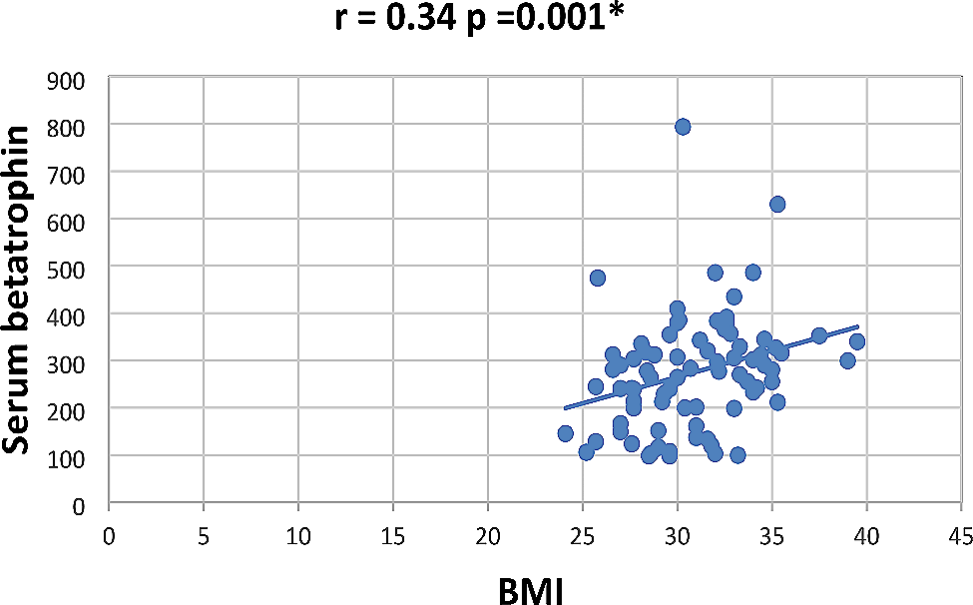
Correlation between BMI and serum betatrophin

**Fig. 3:**
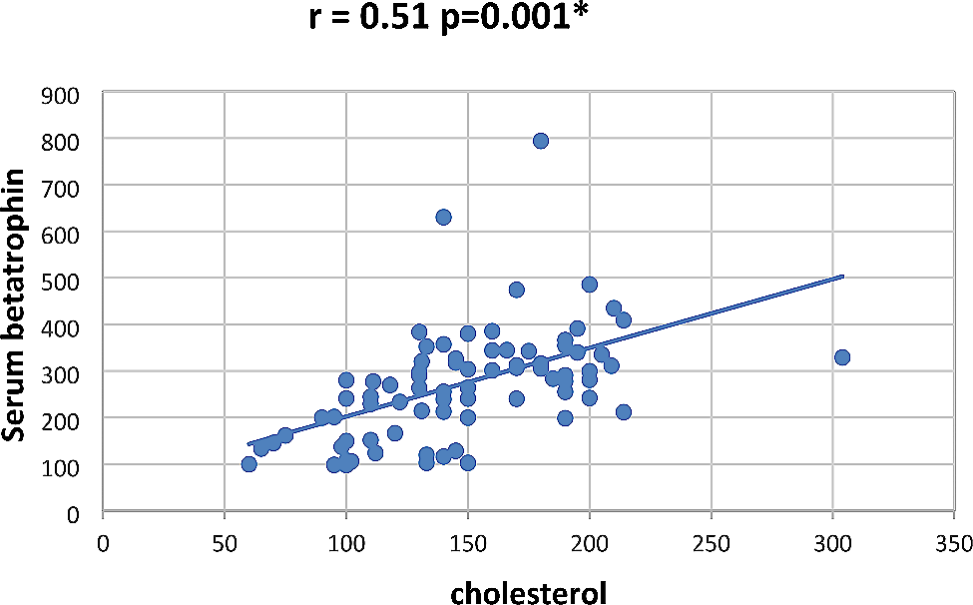
Correlation between cholesterol and serum betatrophin

**Fig. 4:**
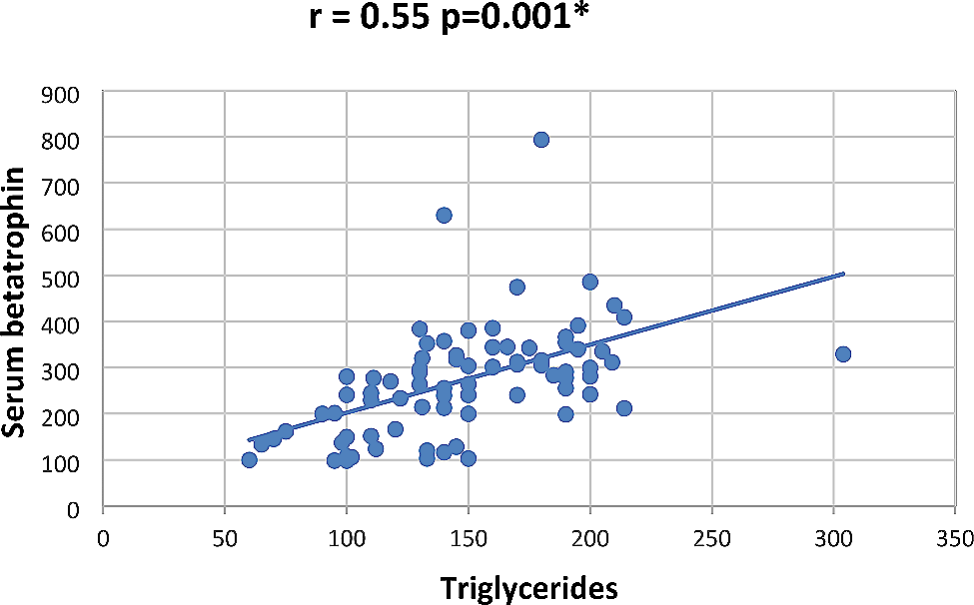
Correlation between triglycerides and serum betatrophin

**Fig. 5:**
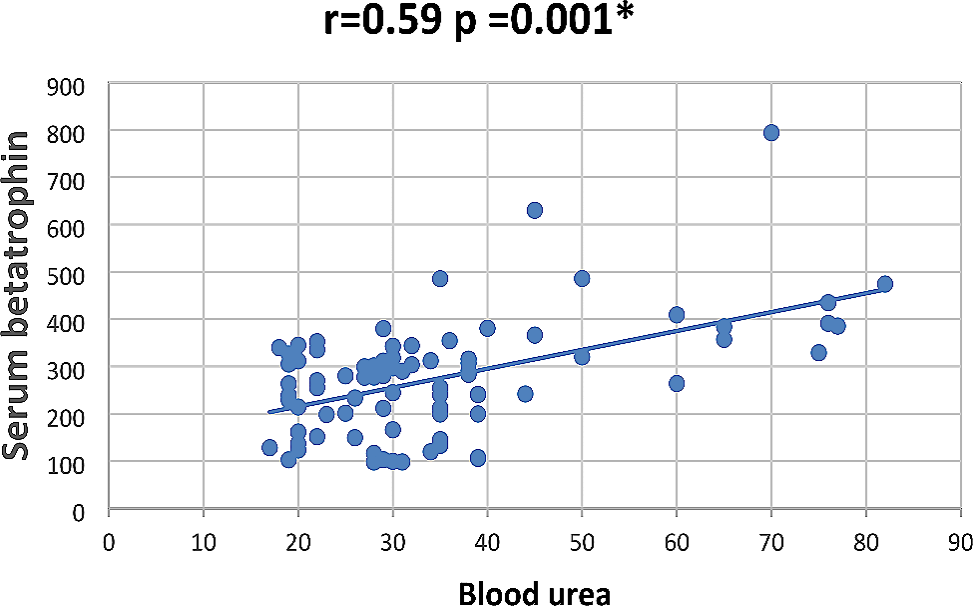
Correlation between blood urea and serum betatrophin

**Fig. 6:**
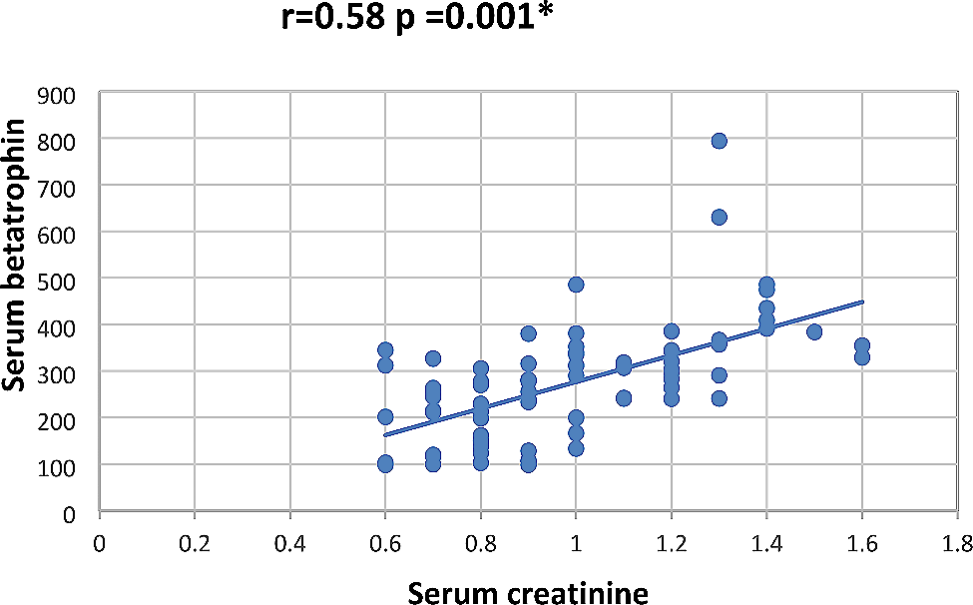
Correlation between Serum creatinine and serum betatrophin

**Fig. 7:**
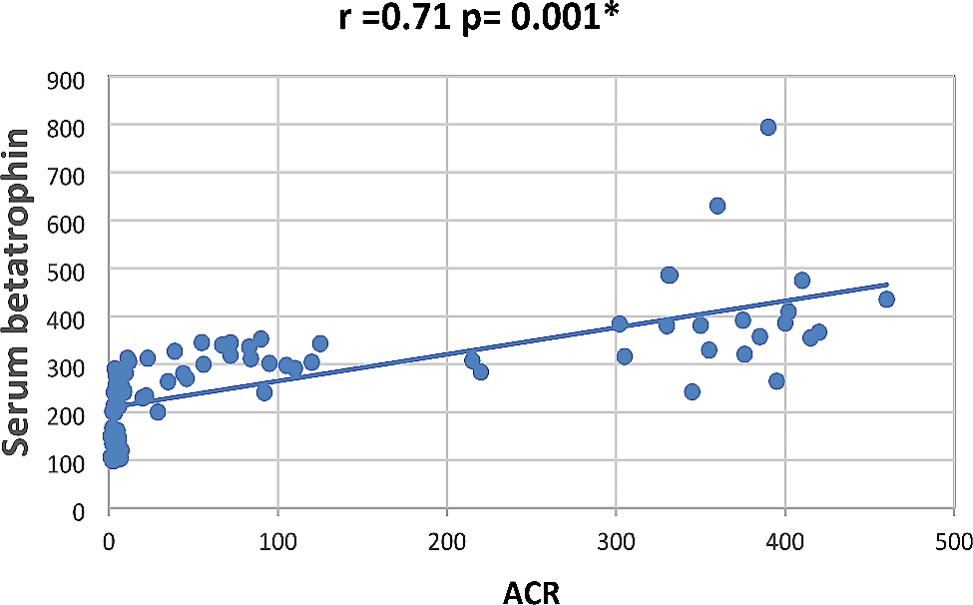
Correlation between UACR and serum betatrophin

**Fig. 8:**
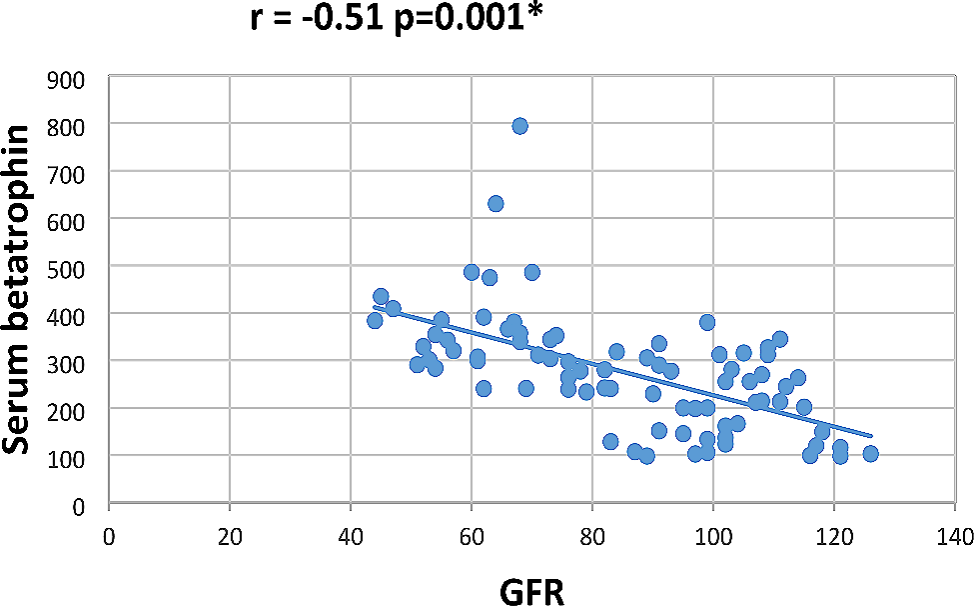
Correlation between eGFR and serum betatrophin

**Fig. 9:**
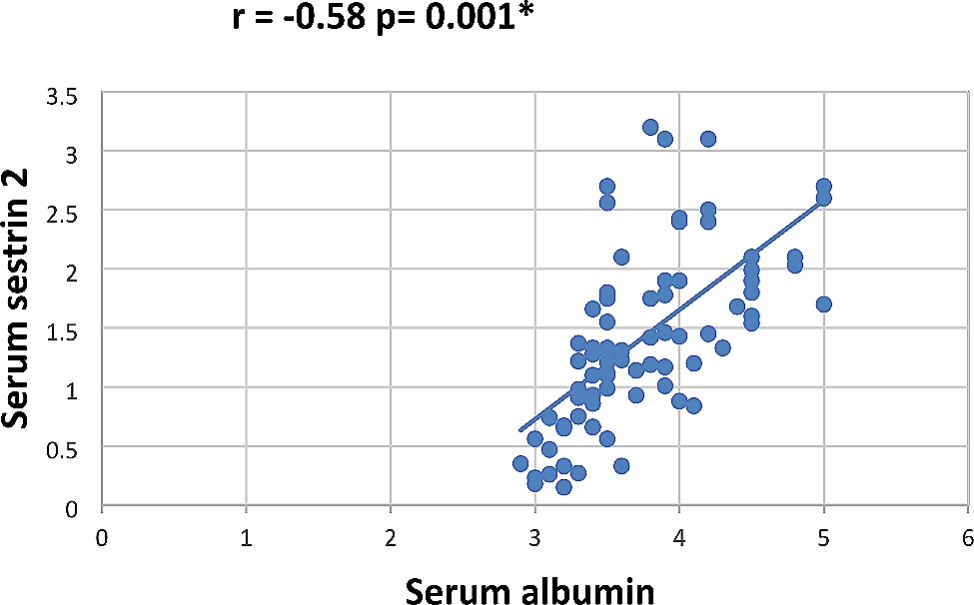
Correlation between serum sestrin 2 and albumin

In our study Pearson correlation between serum Sestrin 2, serum betatrophin and the studied parameters showed that serum betatrophin positively correlated with age, FBG, 2 h PP glucose BMI, TG, total cholesterol, serum creatinine, blood urea, UACR as in Figs. 2, 3, 4, 5, 6 and 7 and negatively correlated with eGFR and serum albumin (Fig. 8) while serum Sestrin 2 negatively correlated with serum albumin (Fig. 9). All correlations are shown in Table 2.

Using the UACR as the dependent variable and our studied parameters as the independent variables, multiple linear regression analysis was performed. The results showed that serum urea, albumin, betatrophin, and BMI are risk factors or predictors for diabetic nephropathy as shown in Table 3.

**Table 1:** Demographic and laboratory parameters of the studied groups

				Group I (*n* = 20)	Group II					Test of sig. P. value		
					Group A (*n* = 20)	Group B (*n* = 20)		Group C (*n* = 20)				
**Sex**	**Male**	9 (45%)			8 (40%)	6 (30%)		12 (60%)		**0.30**		
	**Female**	11 (55%)			12 (60%)	14 (70%)		8 (40%)				
**Age (years)**	Range		27–49		35–54		35–54		45–57		**0.18**	
	Mean ± SD		44.75 *±* 5.35		46.93 *±* 4.59		46.73 *±* 5.79		49.93 *±* 6.59			
**BMI**	Range		24.10–33.20		25.7–35.3		27.8–35.5		25.8–39.5		0.04 *	
	Mean ± SD		29.12 *±* 2.3		29.42 *±* 3.46		32.9 *±* 3.36		33.8 *±* 7.76			
**FBG** **(mg/dl)**	Range		60.0–92.0		98.0–210.0		102.0–230.0		111.0–230.0		0.001*	
	Mean ± SD		76.4 *±* 9.2		139.8 *±* 32.3		147.4 *±* 35.2		149.8 *±* 36.3			
**2 hs PPG** **(mg/dl)**	Range		99.0–125.0		155.0–295.0		168.0– 356.0		125.0–302.0		0.001*	
	Mean ± SD		111.27 *±* 8.8		204.66 *±* 44.2		225.27 *±* 48.8		214.26 *±* 44.2			
**HBA1C** **%**	Range		-		7–9		7–11		7–11		0.04*	
	Mean ± SD		-		7.25 *±* 1.36		9.06 *±* 1.37		9.76 *±* 1.37			
**Duration of DM in years**	Range		-		2–11		3–12		5–12		**0.51**	
	Mean ± SD		-		6.84 *±* 2.81		8.25 + 2.65		8.35 + 3.25			
**Triglycerides** **(mg/dl)**	Range		45.0–110.0		85.0–240.0		70.0–240.0		95.0–250.0		0.001*	
	Mean ± SD		75.4 *±* 13.2		168.8 *±* 52.3		136.4 *±* 53.2		179.8 *±* 43.3			
**Total Cholesterol** **(mg/dl)**	**Range**		**60.0–150.0**		**100.0–214.0**		**100.0– 209.0**		130.0–304.0		0.001*	
	**Mean ± SD**		**104.27 + 27.8**		**152.66 + 34.2**		**161.27 + 38.8**		179.26 *±* 34.2			
**HB (g/dl)**	**Range**		**11.0–13.0**		**10.0–14.0**		**10.0–13.0**		10.0–13.0		**0.19**	
	**Mean ± SD**		**10.48 + 0.93**		**11.40 + 1.20**		**11.42 + 1.89**		11.62 *±* 1.49			
**Total leucocyte count**	**Range**		**5.0–11.0**		**4.0–12.0**		**4.0–12.0**		4.0–11.0		**0.32**	
	**Mean ± SD**		**7 + 2.76**		**6.90 + 2.6**		**7.27 + 2.5**		7.50 *±* 2.8			
**Platelet count**	**Range**		**165.0–452.0**		**167.0–344.0**		**165.0–454.0**		166.0–456.0		**0.33**	
	**Mean ± SD**		**274.7 + 68.3**		**205.8 + 78.9**		**310.7 + 92.3**		276.8 *±* 84.9			
**Blood urea** **(mg/dl)**	**Range**		**17.0–39.0**		**19.0–39.0**		**18.0–38.0**		29.0–82.0		0.001*	
	**Mean ± SD**		**28.1 + 6.2**		**28.8 + 7.3**		**28.7 + 6.2**		55.8 *±* 13.3			
**Serum creatinine** **(mg/dl)**	Range		0.6-1		0.6–1.3		0.6–1.3		0.9–1.6		0.001*	
	Mean ± SD		0.87 *±* 0.14		0.86 *±* 0.16		1.04 *±* 0.24		1.27 *±* 0.21			
**UACR** **(mg/g)**	Range		1.0–7.5		3.0–29.0		35.0–220.0		302.0–460.0		0.001*	
	Mean ± SD		3.5 *±* 1.9		9.6 *±* 7.4		91.8 *±* 55.9		371.5 *±* 55.9			
**e GFR** **(ml/min/1.73m2)**	Range		83.0 -126.0		69.0–112.0		51.0 -114.0		44.0–105.0		0.001*	
	Mean ± SD		104.02 *±* 12.85		94.08 *±* 12.2		76.02 + 20.8		65.28 *±* 16.2			
**Serum albumin** **(g/dl**	Range		3.5–5.0		3.3–4.8		3.3–4.1		2.9–3.6		0.001*	
	Mean ± SD		4.1 *±* 0.50		4.2 *±* 0.42		3.6 *±* 0.52		3.2 *±* 0.24			
**Serum sestrin 2 (ng/ml)**	Range		1.17–3.2		0.93–2.4		0.75–1.42		0.15–1.11		0.001*	
	Mean Mean ± SD		2.32 *±* 0.55		1.62 *±* 0.36		1.02 *±* 0.18		0.55 *±* 0.29			
**Serum betatrophin** (ng/l)	Range		98.0–201.0		199.0 -312.0		240.0 -355.0		242.0–794.0		0.001*	
	Mean ± SD		132.2 *±* 31.6		251.6 *±* 36.26		307.20 *±* 31.16		409.2 *±* 115.32			

Data are presents as mean ± SD, *p* values for ANOVA test, Sig. bet. groups were done using Post Hoc Test (LSD). * Statistically significant at *p* ≤ 0.05

**Table 2:** Pearson correlation between serum sestrin 2, betatrophin and studied parameters of the studied groups

Cases	Serum Sestrin 2		Serum betatrophin	
	*R*	*P*-value	*R*	*P*-value
**Age**	-0.04	0.64	0.63	0.001*
**Fasting blood sugar**	0.14	0.94	0.55	0.001*
**2 h post prandial**	0.01	0.99	0.48	0.001*
**BMI**	-0.07	0.53	0.34	0.001*
**Triglycerides**	-0.06	0.15	0.55	0.001*
**Cholesterol**	-0.08	0.13	0.51	0.001*
**Serum urea**	0.16	0.55	0.59	0.001*
**Serum creatinine**	0.18	0.83	0.58	0.001*
**UACR**	0.11	0.72	0.71	0.001*
**eGFR**	0.36	0.07	-0.51	0.001*
**Serum Albumin**	-0.58	0.001*	-0.58	0.001*

r: Pearson correlation test *: Statistically significant at *P* ≤ 0.05

**Table 3:** Multiple Linear regression of the predictor variables

Variables	Standardized Coefficients Beta	95.0% Confidence Interval		*P*- value
		Lower Bound	Upper Bound	
**Age**	0.166	0.008	0.041	0.167
**BMI**	0.116	0.012	0.069	0.007*
**Triglycerides**	0.051	-0.001-	0.004	0.284
**Cholesterol**	0.072	0.000	0.003	0.131
**Serum urea**	− 0.0142-	-0.020-	-0.001-	0.038*
**Serum creatinine**	0.123	-0.320-	1.423	0.211
**UACR**	0.476	0.002	0.005	0.000*
**eGFR**	-0.002-	-0.009-	0.009	0.983
**Serum albumin**	-0.145-	-0.538-	-0.098-	0.005*
**Serum sestrin 2**	0.051	-0.004-	0.023	0.177
**Serum betatrophin**	0.213	0.001	0.003	0.001*

*: Statistically significant at *P* ≤ 0.05

## Discussion

Diabetes mellitus incidence and prevalence are increasing worldwide because of growing rates of obesity, metabolic syndrome and westernization of lifestyle. Concurrently, the incidence of diabetic kidney disease (DKD) rises; DKD is widely regarded as the most serious complication of DM and a major cause of CKD. Between 20 and 50% of patients with T2DM will eventually develop DKD [21] Sestrin-2 belongs to a highly conserved family of stress responsive proteins with antioxidant characteristics. In order to preserve cellular survival and homeostasis, noxious environmental and metabolic stimuli such as oxidative stress, inflammation and DNA damage induce the expression of Sestrins. Additionally, Sestrin 2 suppresses the production of reactive oxygen species (ROS) [22].

In our study, significant low level of Sestrin 2 was found in the macroalbuminuric group as compared to the other two diabetic and control groups. Although it showed negative correlation with age, BMI, serum cholesterol and TG, it didn’t reach a significant level except for serum albumin. The cut off value of serum Sestrin 2 to predict DN was 0.98 ng/ml with sensitivity 99%, specificity 66%. In multiple linear regression of the predictor for DN where UACR was the dependent variable, Sestrin 2 was a non- significant predictor (*P* value = 0.177).

In hyperglycemic and dyslipidemic environments, Sestrin 2 controls monocyte activation and atherogenic events via the AMPK-mTOR nexus. Sundararajan S, et al., investigated the association between Sestrins levels and dyslipidemia, atherosclerosis and DM. The mRNA expressions of Sestrin1 and Sestrin3 in monocytes in dyslipidemic patients but not in diabetics was significantly reduced. However, Sestrin2 mRNA expression was significantly reduced in diabetics with and/or without dyslipidemia. This finding is consistent with our results. Additionally, glycemic and lipid parameters showed a negative correlation with the levels of sestrin2 mRNA [9].

According to Watany M et al., the degree of albuminuria is correlated with a decrease in Sestrin 2 gene expression and serum protein levels, with macroalbuminuria being associated with the lowest levels. When their results were subjected to multiple regression analysis for prediction of microalbuminuria, it was found that SESN2 expression (*P* value 0.019) but not serum Sestrin 2 (P 0.09) was able to predict it [23]. AMPactivated protein kinase (AMPK) effectively inhibits mTORC1 activity to regulate autophagy. Additionally, AMPK supports mitochondrial activity and has antioxidant characteristics. Sestrin 2 was found to improve the functions of mitochondria in podocytes and apoptosis through AMPK signaling as proved by Lin et al., They investigated the effects of Sestrin 2 on the regulation of AMPactivated protein kinase (AMPK) in streptozotocininduced diabetic rats and DN patients. They revealed that Sestrin 2 expression was decreased in hyperglycemic stimulated podocytes, as well as in diabetic rats and patients with DN [22].

However, in contrast to the healthy control group, Chung et al. observed that obese and T2DM patients had higher serum Sestrin 2 levels. Their results also were positively correlated with BMI, serum TG, glucose, C-reactive protein, and the degree of insulin resistance [12].

One of the angiopoietin family is Betatrophin (MW; 22 KDa); a circulating hormone derived from the liver that stimulates the proliferation of pancreatic β-cells and lipid metabolism. It is also referred to as lipasin, angiopoietin-like protein 8 (ANGPTL8), refeeding induced in fat and liver (RIFL), or hepatocellular carcinoma-associated gene TD26 [14]. Interest in betatrophin as a possible β-cell regenerative treatment for diabetes and subsequent DN has grown. Nevertheless, a number of subsequent investigations suggested that insulin and a high-fat diet may also induce the expression of betatrophin, leading to elevated serum TG and insulin resistance rather than enhanced glucose metabolism [24, 25].

Although type 2 diabetes patients’ resistance to the effects of betatrophin, cannot be ruled out, the results of this study revealed significant high level of betatrophin in the macroalbuminuric group as compared to the other two diabetic and control groups. (*P* value = 0.001) Serum betatrophin showed significant positive correlation with age, fasting, 2 h postprandial, BMI, TG, total cholesterol, serum creatinine, blood urea, UACR, and significant negative correlation with eGFR and serum albumin. Additionally, betatrophin and diabetic nephropathy were found to be significantly correlated using multiple linear regression. (*P* value = 0.001*)

Urinary albumin loss has been associated with insulin resistance, which in turn causes the production of hepatic hormones, that may act as mediators of the increased proliferation of B cells in T2DM. Numerous investigations showed that T2DM patients had elevated betatrophin levels. Chen et al. discovered that T2DM patients had significantly higher serum levels of betatrophin than did healthy subjects, particularly in the macroalbuminuria group. (*P* < 0.001). Serum betatrophin level showed an inverse correlation with eGFR, total cholesterol, and high-density lipoprotein cholesterol (HDL-C), but a positive correlation with sex, diabetes duration, systolic blood pressure (SBP), BMI, UACR, and TG. They concluded that albumin loss may result in increased betatrophin secretion [26] HbA1c, FBG, and TG were found to positively correlate with betatrophin levels by Maurer L et al. Moreover, age, SBP and intima media thickness were all positively correlated with betatrophin while CKD-EPI eGFR was negatively correlated [27].

According to Mohany et al.‘s results, the T2DM group’s serum betatrophin level was higher than that of the healthy control group, and the difference was greater in the macroalbuminuric group. Furthermore, our results were supported by the substantial positive correlations that were discovered in this study between the levels of serum betatrophin and BMI, HbA1C%, glycated albumin%, FBG, and creatinine [10].

In agreement to our results, Alshawaf et al., suggested betatrophin level as a risk predictor for DN in association with angiopoietin 2. The serum levels of Angiopoietin-like proteins (ANGPTL) 3, ANGPTL4, betatrophin, angiopoietin 1 and 2 were measured in 50 type 2 diabetic patients and 67 DN patients compared to 117 healthy participants. When Ang2 and ANGPTL8 were combined, the receiver operating characteristic (ROC) analysis yielded an area under the curve (AUC) of 0.77 and an 80.7% specificity for DN prediction [28].

Conversely, Javier Gómez-Ambrosi et al., discovered that betatrophin levels in the blood were much lower in obese people, and even lower in those with impaired glucose tolerance and T2D participants who had a noticeable sexual dimorphism. Furthermore, they showed that the levels of betatrophin in the blood were significantly greater in women than in men [29]. The differences between our findings and those of other researches on the levels of Sestrin 2 and betatrophin may be caused by various immunoassays, sample size or subject differences, particularly in terms of BMI and diabetes duration.

### Limitations

As a cross-sectional study, the sample size was comparatively small.

## Conclusions

In diabetic nephropathy patients especially macroalbuminuric, the serum level of betatrophin was significantly increased as compared to the other two diabetic and control groups. Serum betatrophin had significant positive correlation with age, fasting, 2 h postprandial, BMI, TG, total cholesterol, serum creatinine, blood urea, UACR, and significant negative correlation with eGFR and serum albumin. Consequently, DN can be significantly predicted by serum betatrophin levels. On the other hand, although serum Sestrin 2 level was significantly decreased especially in macroalbuminuric patients it was not a significant predictor for DN. However, restoring its function could be a new therapeutic target to decrease ROS accumulation and improve autophagy in diabetic nephropathy.

## Data Availability

The datasets used and/or analysed during the current study available from the corresponding author on reasonable request. Sample size: was calculated by using MedCalc software Version 22.009 package for biomedical research. The criteria used for sample size calculation were as follows: •Two-sided confidence level 95%, •Power 80%. •Two study groups, ratio is 1:3 •The outcome (the expression mean?+?SD of serum sestrin2) among type 2 diabetic patients is expected to be 4.1+2.3 compared to 5.9+1.9 among controls according to the results of Mohany et al., 2020. The sample size based on the previously mentioned criteria was found to be 18 participants per group and that number will be increased by (10.0%) to be 20 per group to overcome the drop out rate.
